# Paediatric orbital alveolar soft part sarcoma recurrence during long-term follow-up: a report of 3 cases and a review of the literature

**DOI:** 10.1186/s12886-020-1312-x

**Published:** 2020-02-21

**Authors:** Yujiao Wang, Baixue Du, Mei Yang, Weimin He

**Affiliations:** Department of Ophthalmology, Ophthalmic Laboratory, West China Hospital, Sichuan University, Chengdu, 610041 China

**Keywords:** Alveolar soft part sarcoma, Orbital tumour, Tumour recurrence, Histopathology, Differential diagnosis

## Abstract

**Background:**

Alveolar soft part sarcoma (ASPS) is a clinically and morphologically distinct malignant soft tissue tumour. It occurs mostly in the lower extremities in adults. The purpose of our study was to describe the related clinicopathologic factors, treatment and prognosis of recurrent orbital ASPS in children.

**Case presentation:**

Three children aged from 1 to 12 years presented with unilateral proptosis, restricted ocular motility and impaired visual acuity of the affected eye. Periodic acid Schiff (PAS) -positive diastase-resistant crystalline granules were found in all cases. Immunostaining of TFE-3, INL1 and Ki67 was positive in the tumour cells of two patients. The time until local recurrence of primary tumor in patient 2 and patient 3, who only underwent tumour resection, was shorter than that of patient 1, who underwent tumour excision and postoperative radiotherapy. The recurrent masses were successfully treated with complete tumour excision followed by adjuvant radiotherapy. Patient 1 presented metastasis at 11 years after radiotherapy.

**Conclusions:**

Orbital ASPS in children is easily misdiagnosed due to its rare occurrence and atypical clinical findings. Early diagnosis with multidisciplinary, complete surgical resection combined with adjuvant radiotherapy is essential for achieving long-term disease-free survival in orbital ASPS patients.

## Background

Alveolar soft part sarcoma (ASPS) is an extremely rare soft tissue sarcoma that accounts for 0.5–1% of all sarcomas [1] and has unique histological features, an uncertain cell of origin and variable clinical behaviour. The tumour mostly occurs in adolescents and young adults, and females are more often affected [2]. It usually involves the muscles and deep soft tissue of the extremities, trunk, head and neck, whereas in children, it primarily affects the head and neck regions [3, 4]. As ASPS is a rare orbital tumour and relapses easily, ophthalmologists and pathologists should be aware of the related clinicopathologic features and the potentially aggressive nature of the disease for the early implementation of available treatment options. We reported three children with recurrent orbital ASPS and briefly reviewed the literature.

## Case presentation

After review of the diagnoses of cases accessioned in the database of the Ophthalmology Department and Pathology Department of West China Hospital of Sichuan University, three cases of orbital alveolar soft part sarcoma (ASPS) diagnosed between 1993 and 2019 were identified. The clinical histories, manifestations and pathologic findings of the tumours were reviewed. A literature review was performed using MEDLINE for studies published between 1950 and the present, and EMBASE was searched for studies published between 1980 and the present.

### Case 1

**Fig. 1:**
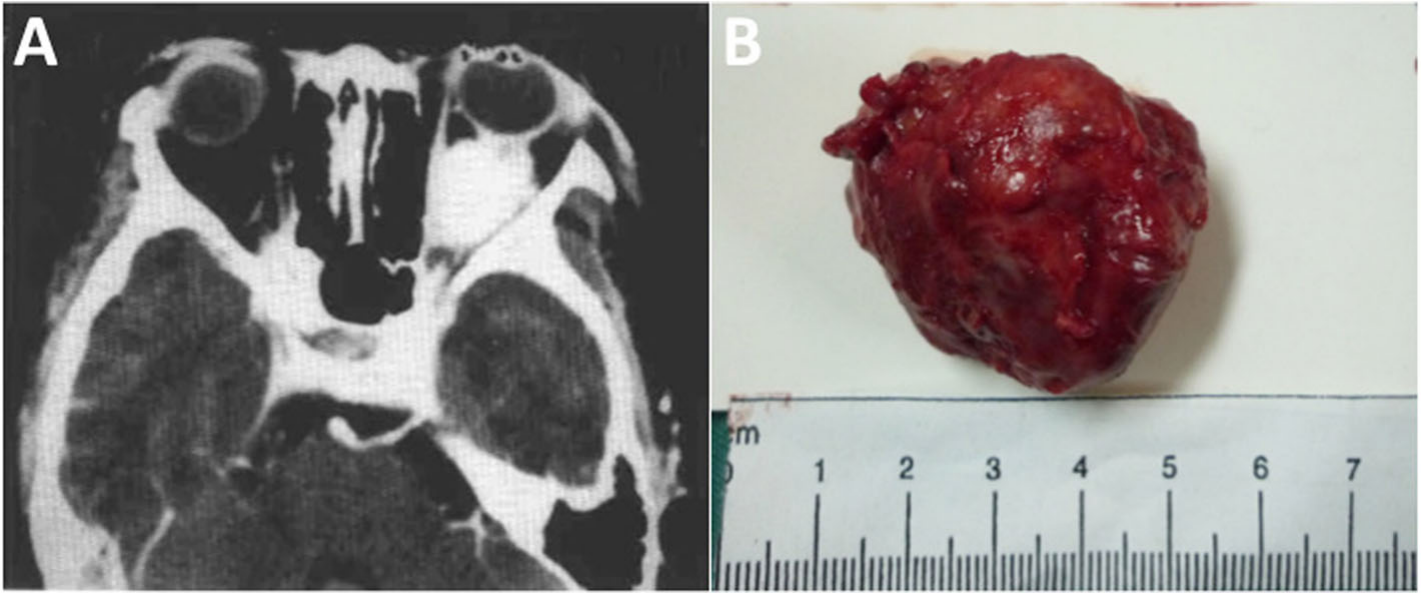
**a** Axial CT scan of the orbits in patient 1, showing a heterogeneous mass surrounding the optic nerve in the posteroinferior orbit. **b** Left orbital mass resected from patient 2

A 9-year-old Chinese girl sought treatment for a five-month history of prominence of the left eye. On examination, the corrected visual acuity was 20/20 OD and 20/25 OS. The left eye had a nonpulsatile and nonreducible proptosis of 8 mm, as revealed by a Hertel exophthalmometer. A dilated epibulbar vessel on the temporal aspect of the left eye and dilated retinal veins of the left fundi were noted by indirect ophthalmoscopy. CT imaging indicated a 3.5 × 2 × 2.5 cm well-defined soft tissue mass occupying the posteroinferior part of left orbit in close relation to the optical nerve that did not invade the orbital wall (see Fig. 1. a). Left lateral orbitotomy showed an intraconal mass surrounding the optic nerve and adherent to neighbouring tissue in the temporal part of the inferior orbit. Tumour excision was performed to achieve a negative surgical margin whenever possible, and the success of this was examined histologically at the point closest to the resected specimen. The pathological diagnosis was ASPS. Subsequently, the girl was scheduled for adjuvant radiotherapy. Seven years later, she returned with gradual progressive painless proptosis of the left eye for eight months. On examination, there was a slight limitation of the left gaze and a 4 mm proptosis of the left eye. CT imaging showed an enhancing retrobulbar tumour of 1.3 × 1.0 × 1.0 cm impinging on the left lateral rectus. The mass was completely resected, and the pathologic diagnosis was recurrent ASPS. The patient underwent regular adjuvant radiotherapy (50 Gy in 15 fraction) for two months. At 7 months after radiotherapy, she did well, with no evidence of metastasis or recurrence. At nearly 11 years, the patient died of cerebral metastases, as reported by telephone follow-up.

### Case 2

A 12-year-old girl presented left upper eyelid swelling and prominence of the left eye for one month. Her corrected vision was 20/20 OD and 20/40 OS. The left eye showed a proptosis of 3 mm with limited motility in all directions. The remainder of the ophthalmic examination results were normal. CT imaging revealed a hyperdense, well-defined intraconal mass of 2.4 × 2.0 × 1.5 cm that compressed the posterior of the eyeball and the superior part of the optic nerve. Although the mass was resected by left orbitotomy and pathologically confirmed to be ASPS, the patient returned with painless prominence of the left eye two months later, indicating local recurrence. Examination of the left eye showed marked eyelid swelling, chemosis and a proptosis of 4 mm with limited motility. A CT scan revealed a tumour of 4.2 × 4.0 × 2.8 cm. After adequate surgical removal tumour (see Fig. 1. b), the pathologic diagnosis of the tumour revealed ASPS recurrence. At that point, the girl underwent regular adjuvant radiotherapy (60 Gy in 20 fraction). At 3 years, she was alive, with no evidence of recurrence or metastasis. Subsequently, she was lost to follow-up.

### Case 3

**Fig. 2:**
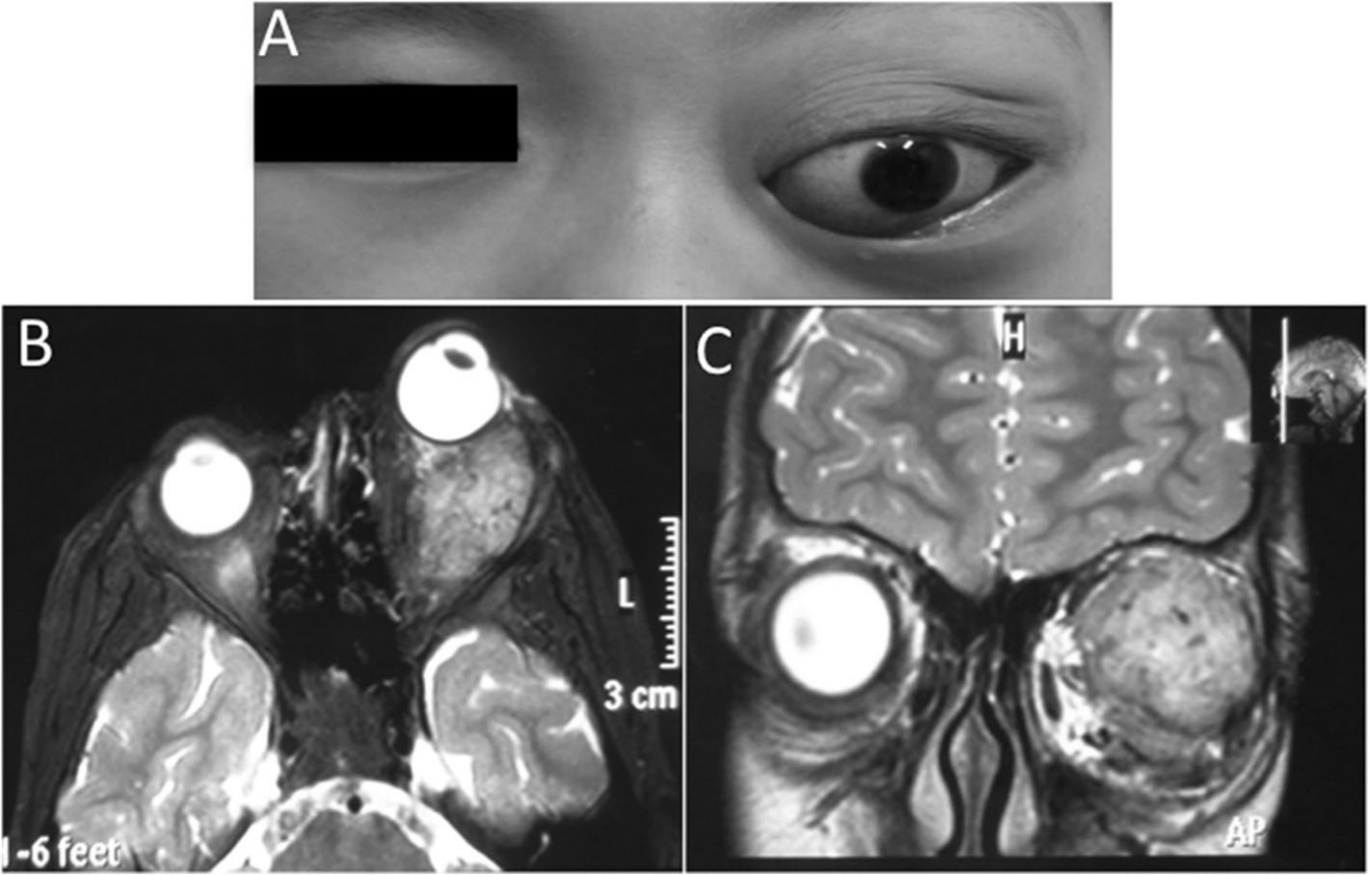
**a** Nonaxial proptosis of the left eye in patient 3, with downward displacement of the globe; **b** and **c**, T2-weighted axial and coronal series showing a well-defined recurrent mass with intermediate signal intensity in the posterosuperior orbit

Five months before admission to the hospital, a 1-year-old boy was noticed to have eyelid swelling accompanied by diminished ocular movements of his left eye. On examination, there was a left proptosis of 4 mm as well as redness and conjunctiva. Ocular motility was restricted on abduction. MRI revealed a hyperdense, extraconal mass of 3.8 × 2.5 × 1.6 cm that was located in the posterosuperior part of the left orbit in close proximity to the optic nerve and lateral rectus and that compressed the lateral eyeball (see Fig. 2). The right eye was normal. After locally and completely excising the mass together with the adjacent soft tissue, the histopathologic diagnosis was ASPS. His parents refused post-surgical radiotherapy, and the patient was then lost to follow-up. Eight years later, the parents sought treatment for a five-year history of prominence of the left eye after the first surgery. Our examination showed a left proptosis of 3 mm with limited ocular motility in all directions. MRI revealed a 4.2 × 2.7 × 3.5 cm retro-ocular mass. The tumour was excised by left orbitotomy, and the pathologic diagnosis indicated ASPS recurrence. The patient was scheduled for (60 Gy in 20 fraction) adjuvant radiotherapy in light of the close surgical margins. He remains under follow-up and is free of recurrence or metastasis.

### Pathologic findings

**Fig. 3:**
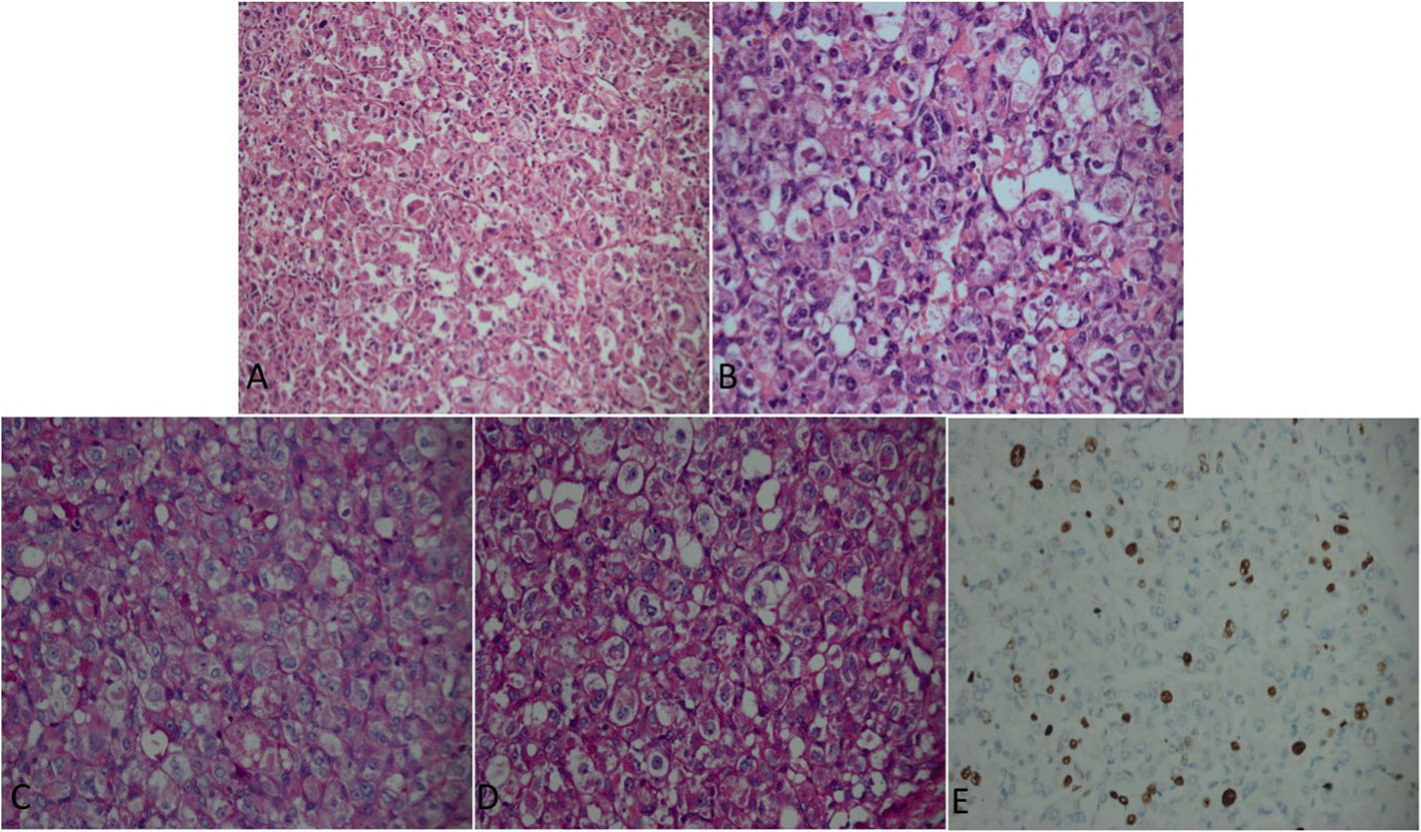
**a** Photomicrograph of the tumour section showing nests of large polygonal cells separated by thin fibrovascular septae (haematoxylin-eosin, original magnification × 200). **b** The cells are large, uniform, and epithelioid with fine, eosinophilic, granular cytoplasm and eccentrically located nuclei containing prominent nucleoli (haematoxylin-eosin, original magnification × 400). **c** PAS revealing characteristic diastase-resistant crystals that vary in size and shape within the cytoplasm of the tumour cells (PAS, original magnification × 400). **d** D-PAS-positive crystalline structures in tumour cells showing non-glycogen materials (D-PAS, original magnification × 400). **e** Ki67 immunostaining of tissue demonstrating strong nuclear immunoreactivity in some tumour cells (Ki67, original magnification × 400)

**Fig. 4:**
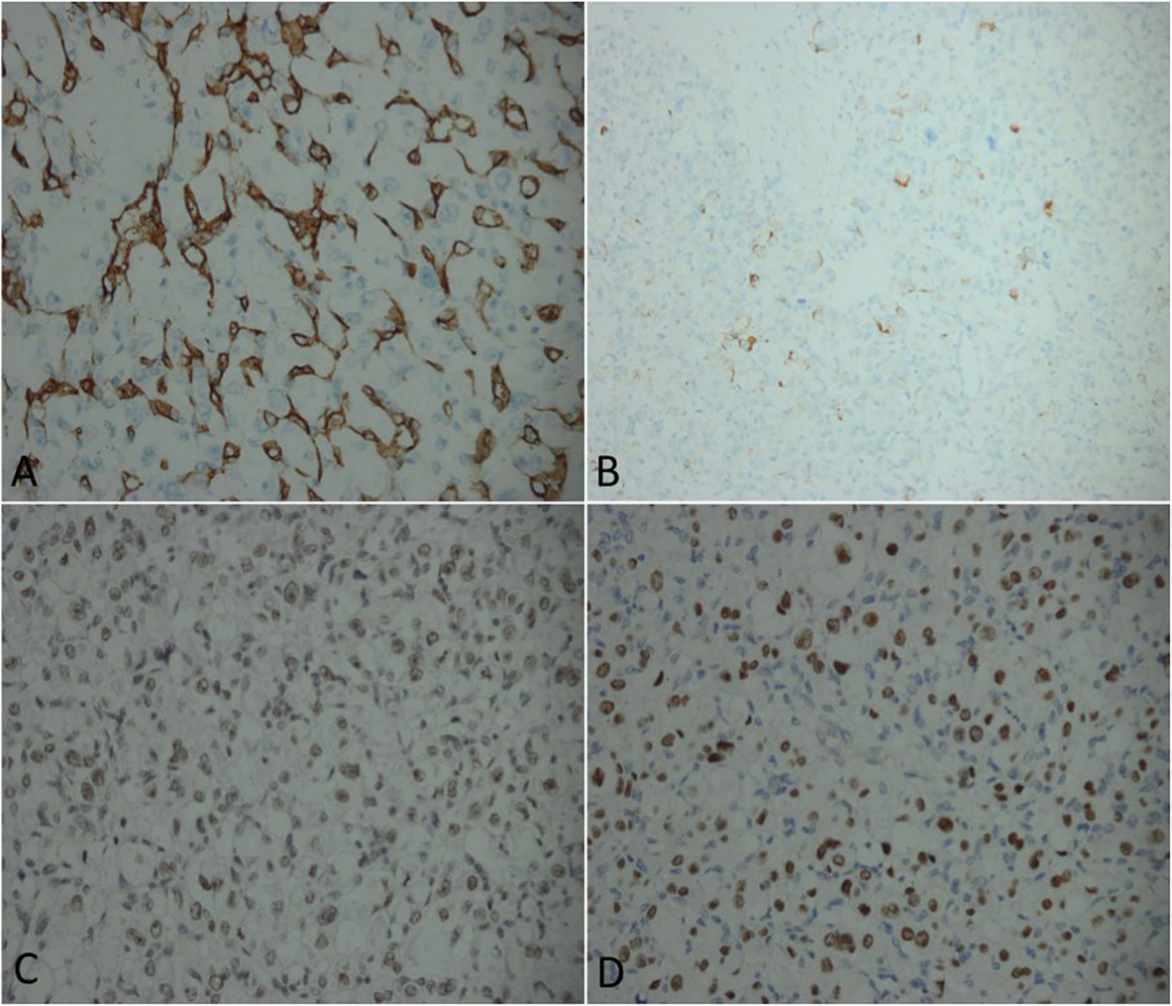
**a** CD34 immunostaining of specimens showing diffuse cytoplasmic immunoreactivity in tumour cells (CD34, original magnification × 400). **b** Desmin immunostaining of tumour cells revealing cytoplasmic positivity (Desmin, original magnification × 400). **c** INL-1 staining of tumour cells showing nuclear immunoreactivity (INL-1, original magnification × 400). **d** Some tumour cells of the specimen show nuclear immunoreactivity after TFE-3 staining (TFE-3, original magnification × 400)

Gross specimens of the tumour appeared yellow to brown in patient 1 and red brown in both patient 2 and patient 3. The specimens were all irregularly shaped soft to firm masses. Microscopic examination of the primary and recurrent tumours from three cases showed that the large oval or polygonal tumour cells were arranged in an organoid or alveolar pattern separated by delicate septa of connective tissue containing capillary-sized vascular channels **(**Fig. 3**.** a). The tumour cells had a distinctly epithelioid appearance with well-defined cell borders, abundant eosinophilic cytoplasm and large vesicular nuclei with prominent nucleoli **(**Fig. 3**.** b). Periodic acid Schiff (PAS) staining revealed characteristic diastase-resistant crystalline structures that varied in size and shape within the cytoplasm **(**Fig. 3**.** c-d). Nuclear atypia and mitotic figures were seen in the tumour cells of the recurrent ASPS in patient 2. Muscle markers (desmin, vimentin, myoglobin, myogenin, MyoD1, and SMA) and non-muscle markers (S-100, TFE-3, INL1, CD34, CD31, EMA and Ki67) were used for immunohistochemical staining in our study **(**Fig. 3-4**)**. The tumours from patient 1 were positive for S100 and myoglobin. TFE-3, INL1 and Ki67 stained positive in patient 2. The tumours of patient 3 were positive for TFE-3, INL-1, CD34, Desmin, and Ki67(ranging from 10 to 20% positivity), indicating the proliferative potential of ASPS.

## Discussion and conclusions

ASPS is a malignant soft tissue sarcoma that rarely occurs in the orbit. A review of the literature indicates 60 reported cases in the orbit to date [4–29]. Orbital ASPS occurs in young patients between 15 and 35 years and has a 3.25:1 female preponderance. The location of ASPS in children is more common in the head and neck regions, especially in the orbit. The cases of orbital ASPS we reported here all occurred in children less than 12 years old, including two girls and a boy.

ASPS usually presents as a slow-growing, painless tumour mass. Orbital lesions have no typical characteristics in terms of clinical and imaging features. ASPS commonly presents proptosis, lid swelling, and dilation of conjunctival vessels. Some patients show compression of the optic nerve, as was seen in our cases, which might lead to impaired vision. PAS staining reveals the characteristic diastase-resistant crystalline structures in ASPS, and D-PAS staining was also used to demonstrate that the PAS-positive structures were not glycogen. Although orbital ASPS has characteristic histopathology, its immunohistochemical staining pattern has been controversial, and the histogenesis of ASPS remains uncertain. Some muscle markers (desmin, vimentin, myoglobin, and MyoD1) and non-muscle markers (S-100) have been reported to be expressed at variable frequencies in ASPS. Our report showed that TFE-3 and INL1 were positive in both the primary and recurrent tumours of two patients. The current concepts demonstrate that ASPS is characterized by the translocation t(X;17) p (11.2;q25), which results in the chimeric ASPSCR1-TFE3 transcription factor, which drives tumorigenesis and provides an important clue for the diagnosis of ASPS [30, 31].

ASPS may be treated as a vasogenic tumour due to its high vascularization on CT imaging, including haemangioma or lymphangioma. The pathologic differential diagnoses of ASPS include paraganglioma, malignant melanoma, alveolar rhabdomyosarcoma, granular cell tumours and metastatic renal cell carcinoma (RCC), which also need to be considered [32]. ASPS usually presents prominent large nucleoli, which are not seen in paraganglioma, which tends to have striking anisonucleosis. Melanoma cells have a better preserved cytoplasm with pigmentation, a clear vacuolated cytoplasm is common in RCC, and alveolar rhabdomyosarcoma cells have a larger nucleus with atypical features and mitotic figures, as compared to ASPS [ 7, 32]. In addition, ASPS is usually negative for epithelial markers (EMA and CK), neuroendocrine markers (synaptophysin and chromogranin A) and melanocytic markers (HMB45 and melan A).

Due to the rare occurrence of orbital ASPS, the optimal management remains controversial. Although complete surgical excision of the small tumour with functional preservation remains the treatment of choice [33], it is difficult to achieve negative surgical margins due to the poorly circumscribed nature of the tumour and its adherence to critical structures such as the optic nerve or extraocular muscles. Local radiotherapy may be beneficial for ASPS by enhancing the local control achieved with limited surgery, by retarding the progression of metastatic deposits, and by providing meaningful palliation [33–35]. Our cases also included small tumours with diameters of less than 5 cm. We performed complete tumour excision followed by local radiotherapy (50–60 Gy in 15–20 fraction) to achieve improved local control and preserved vision. Although the most commonly used chemotherapy agents, such as doxorubicin and ifosfamide, were used in metastatic cases, none have proven to be beneficial [34, 36]. The role of neoadjuvant chemotherapy is also unclear.

The prognosis of ASPS is largely dependent on the initial presentation (localized or metastatic disease), tumour size, and age of onset (patients less than 20 years old may survive significantly longer than those over 20 years old) [37, 38]. Orbital ASPS shows a high incidence of local recurrence in 18.6 to 47.7% of cases. Tumour metastasis might occur late in the course of the disease (median 6 years) and usually appears in the lung, brain, or bone [27, 37, 38]. Interestingly, the two patients that only underwent primary tumour resection presented local recurrence at nearly 2 months and 3 years. Patient 1 had tumour recurrence at 7 years after postoperative radiotherapy. All recurrent masses were successfully treated with tumour resection followed by adjuvant radiotherapy. Patient 1 presented brain metastasis at 11 years after adjuvant radiotherapy. Thus, complete tumour resection and postoperative radiotherapy may offer a better strategy for improving local control. However, we also need more studies to prove the effects of postoperative adjuvant radiotherapy in ASPS. In addition, long-term follow-up is also necessary, as distant metastasis can occur.

## Data Availability

The datasets used and/or analyzed during the current study are available from the corresponding author on reasonable request.

## Abbreviations

ASPS: Alveolar soft part sarcoma
CT: Computed tomography
MRI: Magnetic Resonance Imaging
